# Recurrent giant fibroadenomas with transformation to cystosarcoma phyllodes in a 17-year-old girl: a rare case report from Syria

**DOI:** 10.1186/s13256-019-2313-3

**Published:** 2019-12-21

**Authors:** Sawsan Ismail, Sara Alaidi, Sarah Jouni, Yahya Kassab, Zuheir Al-Shehabi

**Affiliations:** 1 Department of Pathology, Faculty of Medicine, Tishreen University, Lattakia, Syria; 2 Faculty of Medicine, Tishreen University, Lattakia, Syria; 3 Department of General Surgery, Al-Tabiat Surgical Hospital, Lattakia, Syria

**Keywords:** Breast lesions, Fibroadenoma, Phyllodes tumors (cystosarcoma phyllodes)

## Abstract

**Background:**

Fibroadenoma is the most prevalent benign breast lesion that generally affects middle-aged women; it is rare in adolescents and younger children. The transformation into malignancy is not common. However, multiple recurrences of rapidly enlarging fibroadenomas suggest a high possibility of transforming into phyllodes tumors, which are uncommon fibroepithelial lesions that account for 0.3–0.5% of female breast tumors and typically present in premenopausal women.

**Case presentation:**

We report a case of a 17-year-old Syrian girl who previously had three episodes of recurrence of multiple rapidly enlarging fibroadenomas in her left breast and underwent three operations for complete resection of the lesions. However, a few months later, she was readmitted with multiple large masses in the same breast, and pathological findings confirmed a surprising combination of multiple fibroadenomas for the fourth time with a malignant phyllodes tumor (cystosarcoma phyllodes). The patient underwent lumpectomies followed by adjuvant radiotherapy. Long-term follow-up was recommended.

**Conclusion:**

Our patient had an extraordinary number of episodes of recurrence at a young age and a rare combination of malignant and benign lesions in the same breast with multiple recurrences. We present her unique, very challenging case with the aim of highlighting the importance of clinical correlation, detailed diagnosis, and careful follow-up.

## Background

Fibroadenoma (FA) is the most common type of benign breast lesions. It generally affects middle-aged and premenopausal women, whereas it is rare in adolescents and younger children. This tumor is composed of ductal components surrounded by loose fibrous tissue [1]. Giant FA is a rather rare type that accounts for 0.5–2% of all FAs [2, 3]. The transformation into malignancy is not common. However, multiple recurrences of rapidly enlarging FAs suggest a high possibility of transforming into phyllodes tumors (PTs), which are uncommon fibroepithelial lesions that account for 0.3–0.5% of female breast tumors and typically present in premenopausal women [4, 5]. We report a unique case of a patient with four recurrences of multiple large benign FAs and a distinctive transformation into malignant PT concerning the gross and morphological appearance.

## Case presentation

**Fig. 1:**
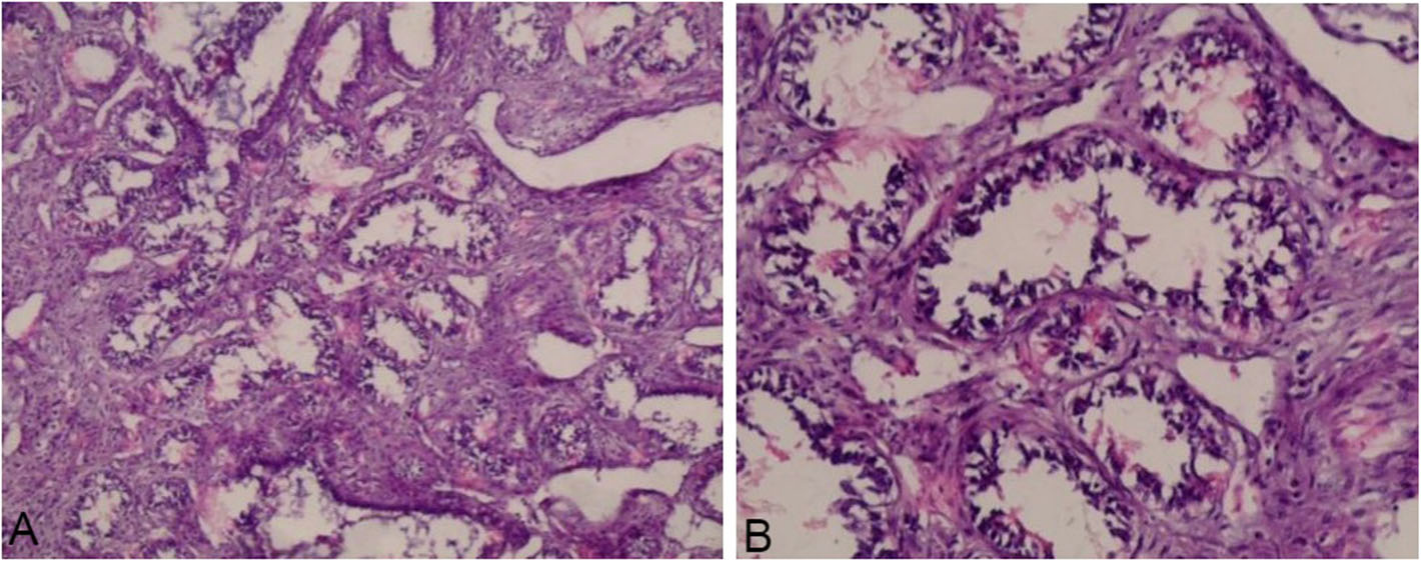
Morphological features of juvenile fibroadenoma: diffuse proliferation of fibrous tissue surrounding acinar and cystically dilated ductal structures with no cellular atypia **a** Hematoxylin and eosin (H&E) stain, original magnification × 200. **b** Hematoxylin and eosin (H&E) stain, original magnification × 400

A 17-year-old Syrian girl presented to our hospital in November 2016 with multiple masses in her left breast. She had no history of anorexia, fever, weight loss, skin ulceration, or nipple discharge. Furthermore, the patient has no siblings, and her family history was insignificant except for diabetes mellitus. On clinical examination, five well-defined mobile masses measuring 9, 8, 5, 3, and 2 cm were present. She did not have axillary lymphadenopathy, and the results of her blood examinations were within normal limits. The breast masses were excised, and microscopically, sections revealed a diffuse proliferation of fibrous tissue, surrounding acinar and cystically dilated ductal structures with no evidence of malignancy within the limits of the specimens (Fig. 1). Accordingly, our patient was diagnosed with multiple juvenile FAs.

One year later, the patient was readmitted to the hospital with multiple breast masses. She had a lobulated mass measuring 2.5 cm in diameter in her right breast, and her left breast had five masses measuring 8, 7, 6, 5, and 4 cm respectively. All six lesions were resected, and morphological examination revealed the diagnosis of multiple cellular FAs with no evidence of cellular atypia. Long-term follow-up of the patient was recommended.

**Fig. 2:**
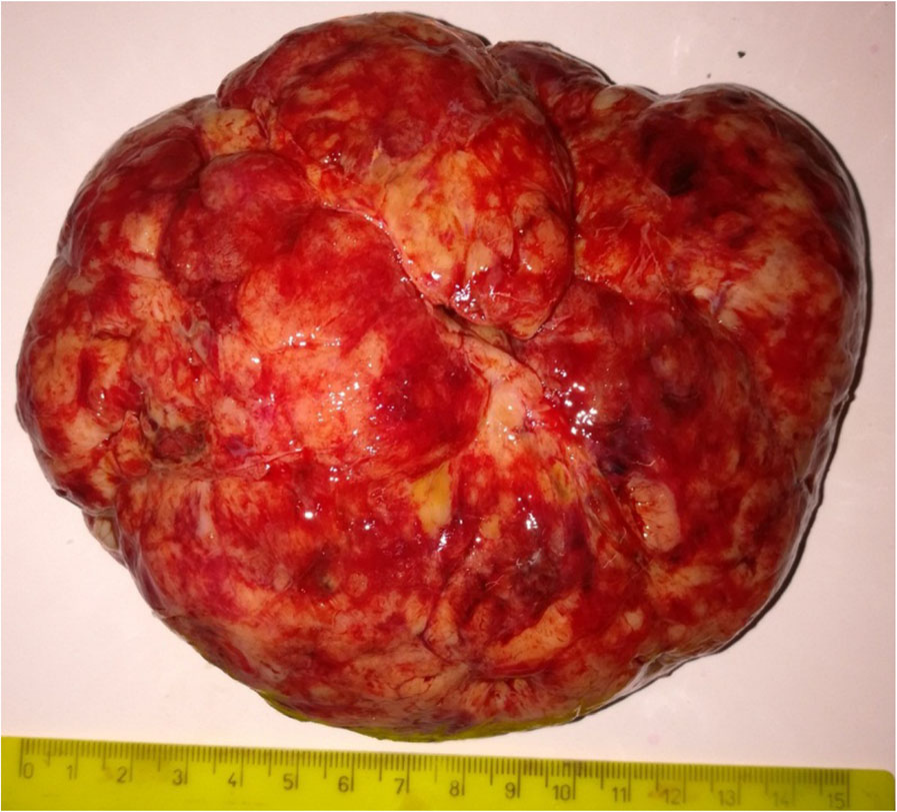
A large, well-circumscribed mass resected in the third recurrence, measuring 15 cm in diameter

**Fig. 3:**
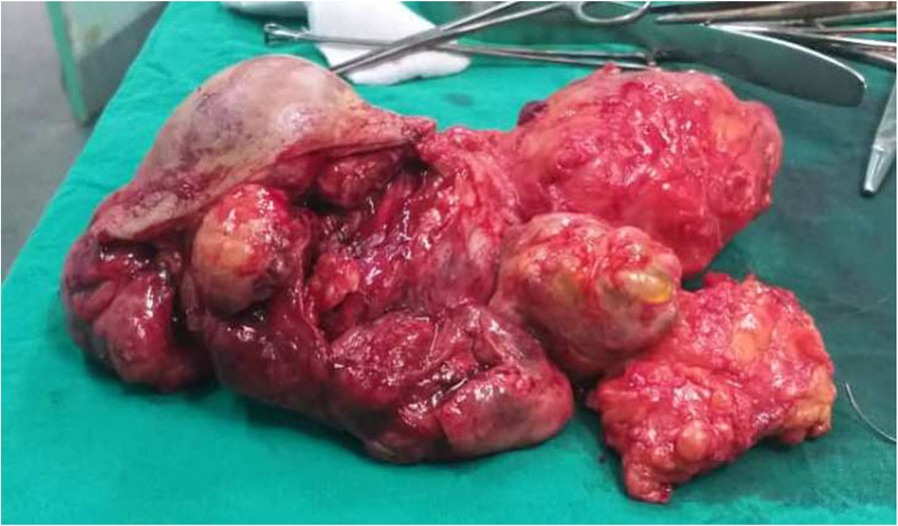
Resected, well-circumscribed masses measuring together 20 × 15 × 16 cm in the fourth recurrence right after the surgical procedure

**Fig. 4:**
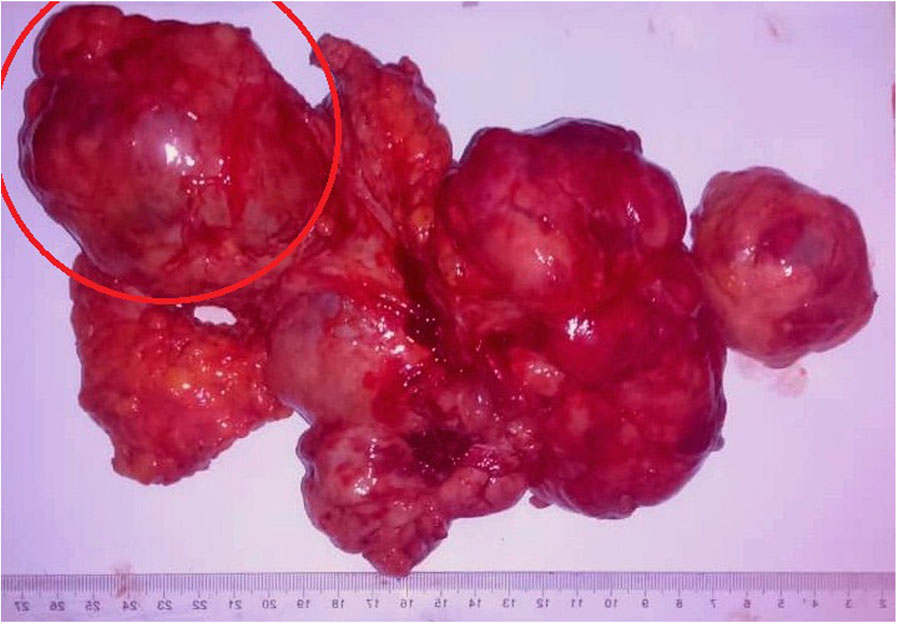
Resected, well-circumscribed masses measuring together 20 × 15 × 16 cm. The *red circle* points to the largest mass, measuring 8 cm in diameter, and diagnosed microscopically as malignant phyllodes tumor

**Fig. 5:**
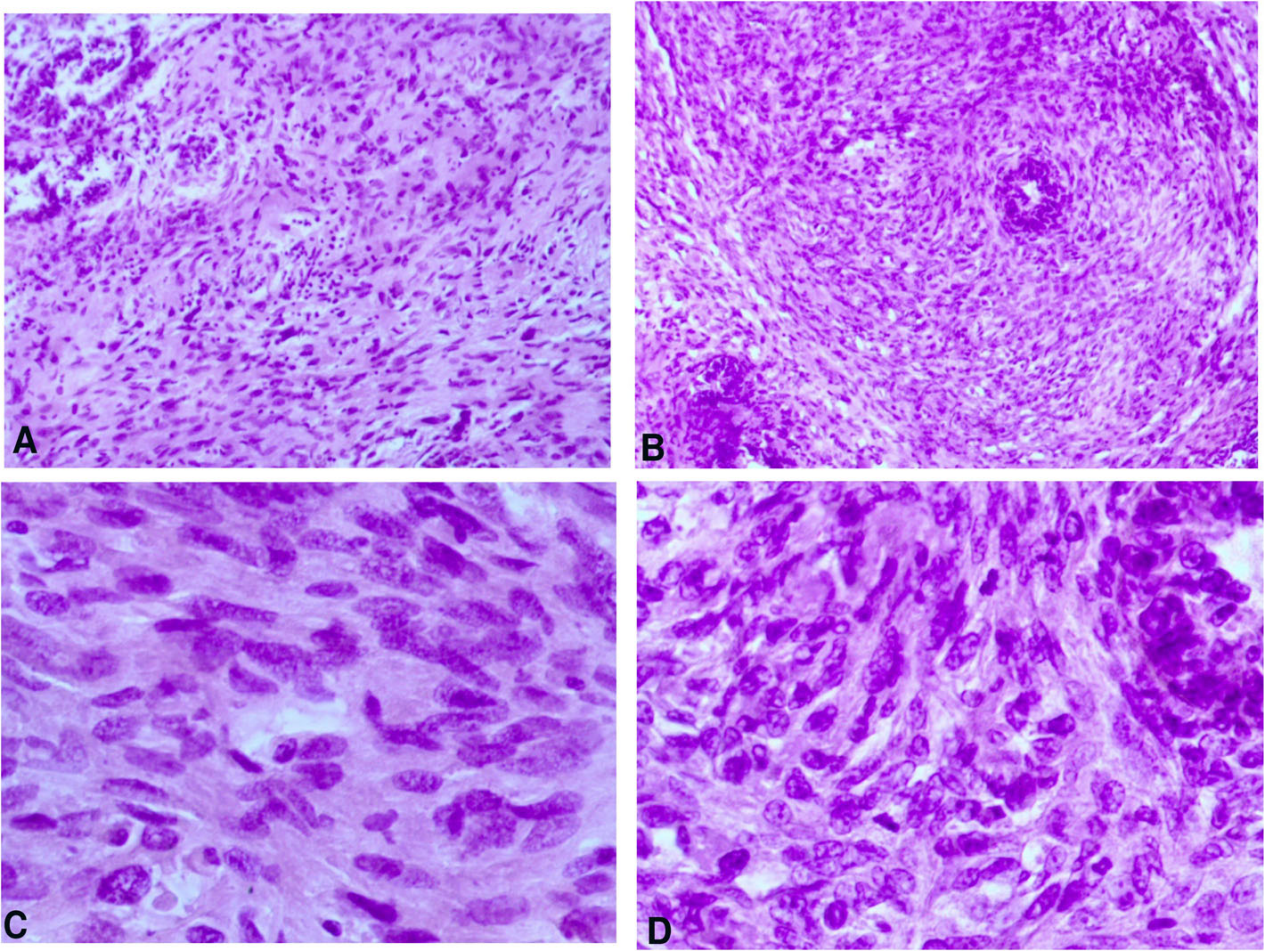
Nodular proliferation of multiplicated cellular fibrous tissue and distended ductal structures with prominent areas of stromal overgrowth, nuclear pleomorphism, and mitotic figures (5–7/10 high-power fields). **a** and **b** Hematoxylin and eosin (H&E) stain, original magnification × 200. **c** and **d** Hematoxylin and eosin (H&E) stain, original magnification × 400

**Fig. 6:**
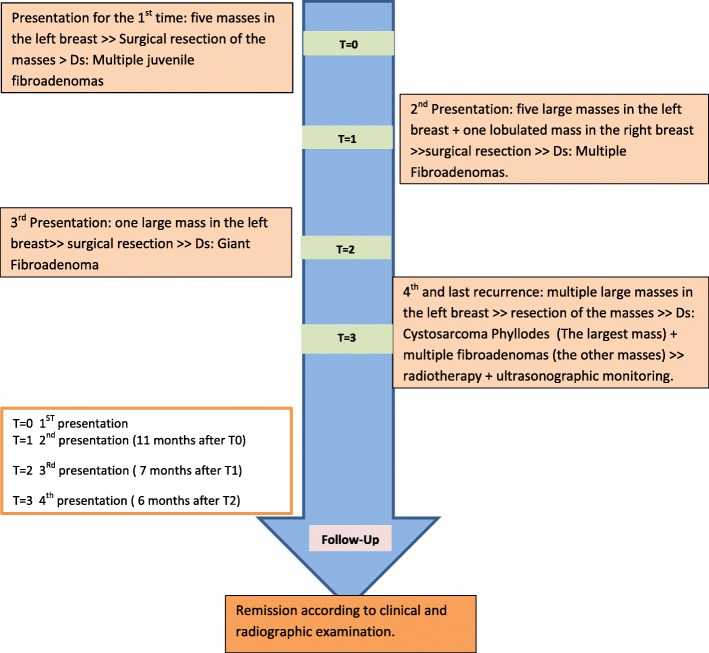
A timeline of the patient’s case. *Ds* diagnosis, *T* time

A few months later, our patient had a new large lobulated mass in her left breast. At this time, her blood examination results were normal, and she had no skin changes. The excisional tumor measured 15 cm in diameter (Fig. 2), and the diagnosis confirmed a giant cellular FA with no cellular atypia. The patient had only 6 months of dormancy before she was readmitted again due to rapid enlargement in the left breast. Clinical examination revealed several lobulated masses, and the patient underwent lumpectomies (Fig. 3). Gross examination revealed multiple masses measuring 20 × 15 × 16 cm (Fig. 4). Interestingly, the largest mass measured 8 cm in diameter, and microscopically it revealed nodular proliferation of multiplicated cellular fibrous tissue and distended ductal structures with prominent areas of stromal overgrowth, nuclear pleomorphism, and occasional mitotic figures (5–7/10 high-power fields) (Fig. 5). As a result, the diagnosis of the largest mass confirmed a high-grade malignant PT (cystosarcoma phyllodes) with microscopic positive foci in the margins, whereas the other lumps were diagnosed as multiple cellular FAs. Computed tomographic images showed no visceral or lymph node metastases. No reexcision was performed, and the patient was referred directly for radiotherapy (50 Gy in 25 fractions) and monitored regularly with ultrasound scanning according to the oncologist’s recommendations. A timeline of the patient’s case is provided in Fig. 6. From her last visit until the initial date of submitting the manuscript of the present report for publication, our patient reported a good general status, and she is still undergoing long-term follow-up. No recurrence has been detected by ultrasound.

## Discussion

FA is the most common benign breast lesion that affects premenopausal women, but it is rare in adolescents and younger children, accounting for only 1% of breast masses [1].

In most cases, FAs present as mobile, painless, solitary masses with distinct borders. However, our patient had four recurrences of multiple giant masses. Ultrasound scanning was used instead of mammography because of the density of breast tissue and the lack of mammographic sensitivity in young patients [6, 7]. Morphologically, sections revealed a cellular proliferation of multiplicated dense fibrous tissue and distended acinar structures lined by one or two layers of cuboid epithelial cells and surrounded by a fibrous capsule [8].

Giant FA is a very rare type that accounts for about 0.5–2% of all FAs and measures > 5 cm in diameter. It usually presents as a large, smooth, firm mass [2, 3]. Although these neoplasms are benign, they can be associated with some morbid manifestations, including venous congestion, pressure necrosis, and ulceration [3]. Recurrence occurs in only 15% of cases of giant FAs [9].

Furthermore, the high number of local recurrences in a short time span in our patient highlights the importance of close follow-up because of the possibility of transformation into malignancy. However, this transformation is rare in most studies, with an incidence of 0.002–0.125% [4]. Even if local recurrence can be a risk factor for malignant transformation, other conditions might play an important role. Some studies indicate that increased stimulation of estrogen might also contribute to this transformation [4]. A very important point is our patient’s previous history of multiple excised FAs in the same breast, which indicates the possibility of transformation into malignancy.

Some observational studies showed a clonal association between metachronous and synchronous FAs and PTs. Hotspot MED12 exon 2 mutations have been shown to contribute to most of these neoplasms, and assays revealed that PTs display higher mutational burdens [10]. However, these studies were not available in Syria, owing to war restrictions.

PTs are rare neoplasms that account for 0.3–1% of all breast lesions with an incidence of 2 per 1 million women annually. They are classified according to stromal overgrowth, cellular atypia, and mitotic activity into benign, borderline, and malignant forms. The malignant form accounts for 30% of PTs and less than 1% of all breast lesions and is known as cystosarcoma phyllodes, even if the term “sarcoma” might refer to high malignancy. However, metastases are rare, and they are mostly found in lungs and bones, whereas the local recurrence rate is about 20% [5].

The distinction between giant FAs and malignant PTs represents a hard challenge because of some histological similarities, including the proliferation of multiplicated dense fibrous tissue and distended acinar structures. However, in PTs, leaflike structures are seen in addition to prominent areas of stromal overgrowth with nuclear pleomorphism and mitotic figures, whereas giant FAs lack these features [5, 11]. Giant FAs must also be differentiated from other lesions, including juvenile breast hypertrophy, giant lipomas, and hamartomas [2].

Juvenile breast hypertrophy lacks the lobule formation found in FAs. Giant lipomas present as encapsulated nodules with adipose tissue. Hamartomas are characterized by a mixture of lobules, ducts, adipose tissue, and fibrous stroma [2]. This accurate diagnosis was made on the basis of histologic examination of the excised specimens because other diagnostic methods, such as fine-needle aspiration cytology, lack reliability and accuracy, whereas core needle biopsy would have had a high risk of bleeding in addition to low diagnostic accuracy [12].

The National Comprehensive Cancer Network (NCCN) recommends wide excision in malignant PTs, and there have not been enough studies confirming the efficacy of adjuvant radiation therapy after margin-negative surgery [13]. However, negative margins were not assessed in our patient, and the pathologist reported focally involved margins. One explanation is that our patient was taken to the operating room for lumpectomies with a presumed diagnosis of multiple FAs due to her previous history. However, it was only in the final pathology that the diagnosis of malignant PT with involved margins was made.

The NCCN recommends consideration of adjuvant radiation therapy for large malignant PTs if negative margins are not obtained, as in our patient’s case, and the surgical management of a local recurrence was not performed due to potential risk of morbidity [13]. Thus, the oncologist recommended the application of adjuvant radiation therapy with long-term follow-up in our young patient with her remarkable history of recurrent surgeries in a short time span.

## Conclusions

Our patient’s very rare case represented a hard diagnostic challenge due to the rarity of presenting at a young age; difficulties in the differential diagnosis; and the combination of juvenile FAs, giant FAs, and malignant PTs with an extraordinary number of relapses. However, with clinical correlation and detailed observation of morphologic findings, we were able to confirm the diagnosis every time.

## Data Availability

Not applicable.

## Abbreviations

FA: Fibroadenoma
NCCN: National Comprehensive Cancer Network
PT: Phyllodes tumor
